# Relationship of Secondary Structures and Wear Resistance of Antifriction Aluminum Alloys for Journal Bearings from the Point of View of Self-Organization During Friction

**DOI:** 10.3390/e21111048

**Published:** 2019-10-27

**Authors:** Iosif Gershman, Alexander Mironov, Pavel Podrabinnik, Ekaterina Kuznetsova, Eugeniy Gershman, Pavel Peretyagin

**Affiliations:** 1 Laboratory of Electric Currents Assisted Sintering Technologies, Moscow State University of Technology “STANKIN”, Vadkovsky lane 3a, 127055 Moscow, Russiap.podrabinnik@stankin.ru (P.P.); evkuznetsova11@gmail.com (E.K.); gershmanei@gmail.com (E.G.); p.peretyagin@stankin.ru (P.P.); 2 Department of scientific research programs, grants and projects, Railway Research Institute JSC “VNIIZHT”, 3rd Mytischinskaya Street 10, 107996 Moscow, Russia

**Keywords:** antifriction aluminum alloys, wear rate, alloy compositions, secondary structures, elemental composition, friction, self-organization

## Abstract

The paper investigates the relationship between the tribological properties/compositions of new aluminum antifriction alloys and compositions of the secondary structures formed on their friction surfaces. Eight alloys with various compositions have been analyzed. The elemental compositions of the secondary structures on their friction surfaces have been determined. The relationship between the alloy secondary structure compositions with wear rate has been found. An attempt has been made to determine the secondary structure composition patterns based on the non-equilibrium thermodynamics and self-organization theory.

## 1. Introduction

This paper continues the studies of new antifriction aluminum alloys beginning in [1,2,3,4,5,6,7,8]. Previously, the authors investigated compositions, mechanical and tribological properties of the new antifriction aluminum alloys. In [1,2] the compositions of the secondary structures and mechanisms of their formation on the friction surfaces of aluminum alloys and steel counter-body were investigated.

Antifriction aluminum alloys have been developed for replacement of antifriction bronzes in journal bearings [4]. Basically, developments of antifriction aluminum alloys aim at increasing the lead and tin contents in the alloys [9,10,11,12]. However, mechanical properties of the alloys could be worsened in that case [13]. Therefore, powder metallurgy is often used to obtain the said alloys [14,15,16,17,18,19,20].

Since the end of the XIX century, it has been argued that the tribological properties of the materials depend on the properties of the secondary structures on the friction surface [21,22,23,24]. There are numerous studies of secondary structures on friction surfaces of various tribosystems, including journal bearings [25,26,27,28,29,30]. The said studies investigated the secondary structure compositions and properties; however, there are no studies investigating their formation patterns. For example, it is not understood why the contents of various alloying elements in the secondary structures may exceed or fall short of their contents in the antifriction alloy and whether the fact is associated with the friction conditions. The papers within the tribological materials science report that formation of the secondary structures results in reduced friction and wear. Therefore, the formation patterns of the secondary structures with various compositions should to be investigated within the studies of the secondary structure compositions and properties. Based on the said studies, further control of the antifriction alloy compositions will be possible.

In [31,32] it was shown that self-organization results in considerably reduced wear rate. In this context, this work is an attempt to find the secondary structure composition patterns based on the non-equilibrium thermodynamics and self-organization theory, provided that self-organization occurs during the run-in and further friction periods. According to [33,34], self-organization is understood as the process of formation of dissipative structures. Dissipative structures are structures that are formed far from equilibrium, characterized by the abrupt appearance of processes with negative entropy production. Before self-organization, these processes did not stably proceed, only as fluctuations. A significant part of the friction energy is expended on the maintenance of dissipative structures. Consequently, part of the friction energy that was spent on wear before self-organization is reduced. Thus, self-organization leads to a noticeable decrease in wear rate.

For searching the formation patterns of the secondary structures on the friction surface of an antifriction material, we apply the non-equilibrium thermodynamics and self-organization theory I. Prigogine [33,34] similarly [15,16]. Considering the fact that according to [31,32], self-organization results in reduced wear rate, we investigate the possibility of self-organization occurrence during the run-in period. As opposed to [15,16] in this paper, time is used as the variable that characterizes the tribosystem deviation from equilibrium. It is related to the fact that formation of the secondary structures and their development occur during the run-in period. Therefore, the secondary structure states depend on the time. We assume that after the run-in period is finished, the secondary structures go into the stationary state, i.e., if the substance and energy flows are available, the parameters and compositions of the secondary structures do not actually change. In this regard, the compositions of the secondary structures are studied after wear tests.

According to [33,34], self-organization is possible only after the system has lost its thermodynamic stability. Therefore, let’s consider the conditions of the highest probability for the tribosystem to lose its thermodynamic stability.

The expression for the entropy production ($\frac{{\mathrm{dS}}_{i}}{\mathrm{dt}}$) with the assumption that friction only acts in the tribosystem is shown in (1):

(1) $$\frac{{\mathrm{dS}}_{i}}{\mathrm{dt}}=X_{f}J_{f}=\frac{{\left(\mathrm{kpv}\right)}^{2}}{{\lambda T}^{2}},$$

where: $J_{f}$—friction-caused heat flow into the rubbing body equal to −λgradT=kpv; $X_{f}$—thermodynamic force that causes heat flow, when friction occurs equal to $\mathrm{gradT}/T^{2}$; k—coefficient of friction; p—pressure in contact; v—sliding velocity; λ—coefficient of thermal conductivity.

Equation (1) is made under the assumption that friction is the only source of energy in the system. It is believed that more than 90% of the friction energy is converted into thermal energy. Therefore, in equation (1) it is assumed that the main energy dissipation occurs due to thermal conductivity. The remaining processes occurring in the surface layers during friction, we tried to take into account the change in the coefficients of friction and thermal conductivity of the surface layers with time.

In [33,34] for the conditions of the thermodynamic stability loss, the second variation of entropy is proposed to be used as the Lyapunov function $\delta^{2}s$. The thermodynamic stability of the system is determined by the following condition [33,34]:

(2) $$\frac{\partial }{\partial t}\left(\delta^{2}s\right)\geq 0,$$

At the same time, according to [33,34] the derivative of the second variation of entropy is equal to:

(3) $$\frac{\partial }{\partial t}\left(\delta^{2}s\right)=\sum_{n}{\delta X}_{n}{\delta J}_{n},$$

The value on the right side of (3) is called the excessive entropy production. It follows from (2) and (3) that the system can lose its thermodynamic stability under the condition (4):

(4) $$\frac{\partial }{\partial t}\left(\delta^{2}s\right)<0,$$

With beginning of the run-in period, the tribosystem begins to deviate from the equilibrium state in course of time. The excessive entropy production of the rubbing body, considering (1) will be equal to (assuming that only k and λ change in course of time):

(5) $${\delta X}_{f}{\delta J}_{f}=\delta \left(\mathrm{kpv}\right)\delta \left(\frac{\mathrm{kpv}}{{\lambda T}^{2}}\right)=\frac{{\left(\mathrm{pv}\right)}^{2}}{T^{2}}\left(\frac{1}{\lambda }{\left(\frac{\partial k}{\partial t}\right)}^{2}-\frac{k}{\lambda^{2}}\frac{\partial k}{\partial t}\frac{\partial \lambda }{\partial t}\right){\left(\delta t\right)}^{2},$$

The excessive entropy production in (6) may become negative, provided that:

(6) $$\frac{\partial k}{\partial t}\frac{\partial \lambda }{\partial t}>0,$$

The condition (6) is observed when the coefficient of friction and thermal conductivity of the surface layers simultaneously decrease or simultaneously increase in the run-in process. Simultaneous increase in the coefficient of friction and thermal conductivity is typical for seizure prevailing in the run-in process while the secondary structures are not formed. Simultaneous decrease in the coefficient of friction and thermal conductivity is typical for formation of the secondary structures without seizure.

Let us consider the conditions required for self-organization occurrence in the run-in process which correspond to the conditions of the thermodynamic stability loss. For the purpose, the processes that occur in the friction zone without seizure, in the friction zone with seizure and mass transfer in the zones will be considered. The expression for the entropy production in that case is shown in (7):

(7) $$\frac{{\mathrm{dS}}_{i}}{\mathrm{dt}}=\sum_{i}X_{i}I_{i}=\left(\mathrm{kpv}\right)\frac{\mathrm{kpv}}{\lambda \mathrm{TBn}}+\sigma \frac{v\left(1-n\right)B}{T}+X_{s}\frac{\rho_{s}w_{s}\left(1-n\right)B}{T}+X_{f}\frac{\rho_{f}w_{f}\mathrm{nB}}{T},$$

where: B—contact area, n—portion of contact area without seizure, 1−n—portion of contact area with seizure, σ—average strength of seizure bridges, $\rho_{s}$—density of substance being preferably transferred into friction zone with seizure, $X_{s}$—thermodynamic force that causes mass transfer into friction zone with seizure, $w_{s}$—velocity of mass transfer into friction zone with seizure, $\rho_{f}$—density of substance being preferably transferred into friction zone without seizure, $X_{f}$—thermodynamic force that causes mass transfer into friction zone without seizure, $w_{f}$—velocity of mass transfer into friction zone without seizure.

On the right side of expression (7), the first and second terms characterize the entropy productions in the friction zones without seizure and with seizure, respectively; while the third and fourth terms characterize the entropy productions resulted from mass transfer in the friction zones without seizure and with seizure, respectively, similarly to [35].

Successful run-in usually results in decrease in the coefficient of friction, wear rate, seizure area, temperature, and increase in the contact area [24]. Considering (6), the thermal conductivity of the surface layer shall reduce. Those run-in results are achieved due to formation of the relevant secondary structures. However, on the initial run-in stage when the equilibrium oxide films are destructed, the coefficient of friction, wear rate, seizure area, temperature in the contact, and thermal conductivity of the surface layers increase [36]. On the initial run-in stage, the following conditions are observed:

(8) $$\frac{\partial k}{\partial t}>0,$$

(9) $$\frac{\partial \lambda }{\partial t}>0,$$

(10) $$\frac{\partial B}{\partial t}>0,$$

where: B – area of contact;

(11) $$\frac{\partial n}{\partial t}<0,$$

Behavior of other variables included in (7) in course of time on the initial run-in stage is unknown. Their behavior on the initial run-in stage will be investigated based on the highest probability of the thermodynamic stability loss (condition of possible self-organization). The highest probability of the thermodynamic stability loss will be determined based on the maximum possible number of negative terms in (3).

Let’s consider the probability of the thermodynamic stability loss by the tribosystem with the entropy production (7) when the conditions of (8)–(11) are observed in the run-in process. Excessive entropy production is equal to:

(12) $$\sum_{i}{\delta X}_{i}{\delta I}_{i}=\delta \left(\mathrm{kpv}\right)\delta \left(\frac{\mathrm{kpv}}{\lambda \mathrm{TBn}}\right)+\delta \sigma \delta \left(\frac{v\left(1-n\right)B}{T}\right)+{\delta X}_{s}\delta \left(\frac{\rho_{s}w_{s}\left(1-n\right)B}{T}\right)+{\delta X}_{f}\delta \left(\frac{\rho_{f}w_{f}\mathrm{nB}}{T}\right)\;=\frac{{\left(\mathrm{pv}\right)}^{2}}{\mathrm{BTn}\lambda }({\left(\frac{\partial k}{\partial t}\right)}^{2}-\frac{k}{\lambda }\frac{\partial \lambda }{\partial t}\frac{\partial k}{\partial t}-\frac{k}{B}\frac{\partial B}{\partial t}\frac{\partial k}{\partial t}-\frac{k}{n}\frac{\partial n}{\partial t}\frac{\partial k}{\partial t}-\frac{k}{T}\frac{\partial T}{\partial t}\frac{\partial k}{\partial t}){\left(\delta t\right)}^{2}+\frac{v}{T}(\frac{\partial \sigma }{\partial t}\frac{\partial B}{\partial t}\left(1-n\right)-\frac{\partial \sigma }{\partial t}\frac{\partial n}{\partial t}B\;-\frac{\partial \sigma }{\partial t}\frac{\partial T}{\partial t}\frac{B\left(1-n\right)}{T}){\left(\delta t\right)}^{2}+\frac{1}{T}(\frac{\partial X_{s}}{\partial t}\frac{\partial \rho_{s}}{\partial t}w_{s}\left(1-n\right)B+\frac{\partial X_{s}}{\partial t}\frac{\partial w_{s}}{\partial t}\rho_{s}\left(1-n\right)B\;+\frac{\partial X_{s}}{\partial t}\frac{\partial B}{\partial t}\rho_{s}w_{s}\left(1-n\right)-\frac{\partial X_{s}}{\partial t}\frac{\partial n}{\partial t}\rho_{s}w_{s}B-\frac{\partial X_{s}}{\partial t}\frac{\partial T}{\partial t}\frac{\rho_{s}w_{s}\left(1-n\right)B}{T}){\left(\delta t\right)}^{2}\;+\frac{1}{T}(\frac{\partial X_{f}}{\partial t}\frac{\partial \rho_{f}}{\partial t}w_{f}\mathrm{nB}+\frac{\partial X_{f}}{\partial t}\frac{\partial w_{f}}{\partial t}\rho_{f}\mathrm{nB}+\frac{\partial X_{f}}{\partial t}\frac{\partial n}{\partial t}\rho_{f}w_{f}B+\frac{\partial X_{f}}{\partial t}\frac{\partial B}{\partial t}\rho_{f}w_{f}n\;-\frac{\partial X_{f}}{\partial t}\frac{\partial T}{\partial t}\frac{\rho_{f}w_{f}\mathrm{nB}}{T}){\left(\delta t\right)}^{2})$$

or the thermodynamic stability loss to become possible, the excessive entropy production (12) must satisfy the condition:

(13) $$\sum_{i}\delta X_{i}\delta I_{i}<0,$$

The probability for (12) to satisfy this condition will be estimated based on the ratio of the negative and positive terms on the right side of the expression.

The first member on the right side of (12) comprises 5 terms. Based on the conditions of (8)–(11), 3 terms are negative.

The second member on the right side of (12) comprises 3 terms. Based on the conditions of (8)–(11), maximum 2 terms can become negative, provided that:

(14) $$\frac{\partial \sigma }{\partial t}<0$$

The third member on the right side of (12) comprises 5 terms. Based on the conditions of (8-11), maximum 4 terms can become negative, provided that:

(15) $$\frac{\partial X_{s}}{\partial t}<0;\;\frac{\partial w_{s}}{\partial t}>0;\;\frac{\partial \rho_{s}}{\partial t}>0$$

The fourth member on the right side of (12) comprises 5 terms. Based on the conditions of (8–11), maximum 4 terms can become negative, provided that:

(16) $$\frac{\partial X_{f}}{\partial t}>0;\;\frac{\partial w_{f}}{\partial t}<0;\;\frac{\partial \rho_{f}}{\partial t}<0$$

Therefore, from 18 terms (12) maximum 13 can become negative under the conditions of (8)–(11) and (14)–(16). Observation of (14)–(16) results in the highest probability of the thermodynamic stability loss and self-organization occurrence. It follows from (14)–(16) that for increasing the self-organization probability, relatively heavy elements should be preferably transferred into the friction zone with seizure, while relatively light elements should be preferably transferred into the friction zone without seizure.

In [36] it is shown that the compositions of the secondary structures formed on the initial run-in stage undergo only minor subsequent changes, in spite of changes in sizes and surface layer structures.

## 2. Materials and Methods

**Figure 1:**
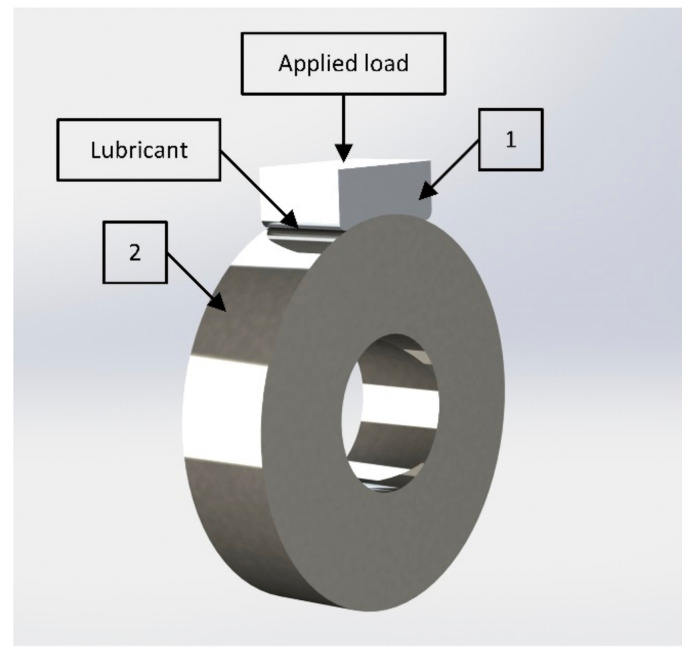
Tribological test scheme. 1—shoe, 2—roller.

The tribilogical tests were carried out based on the shoe-and-roller scheme. The shoe was made of antifriction aluminum alloys while the roller was made of chromium-nickel steel. SNC28 steel (Hakuro Group, Kasai, Japan) was an analogue. The radius of the shoe and roller was 20 mm, while their thickness was 10 mm. The steel composition is shown in Table 1. The tribological test scheme is shown in Figure 1. The steel roller rotated at a speed of 500 rpm. The tests were carried out in the API CB oil. Details of the test procedures are described in [1,2,3].

The shoes were made of antifriction aluminum alloys. The alloy compositions are shown in Table 2.

The secondary structure compositions were determined using the microanalyzer of a scanning electron microscope. The tested area of each friction surface was approximately 100 mm^2^.

## 3. Results and Discussions

**Figure 2:**
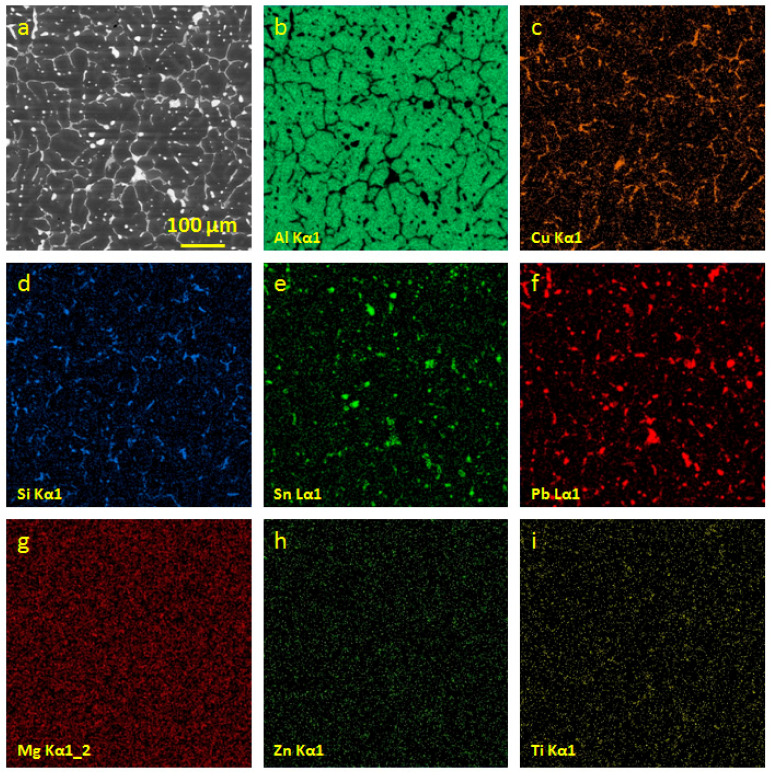
The initial structure of the alloy AO-5.8 (**a**); maps of the distribution of aluminum (**b**); copper (**c**); silicon (**d**); tin (**e**); lead (**f**); magnesium(**g**); zinc (**h**) and titanium (**i**).

**Figure 3:**
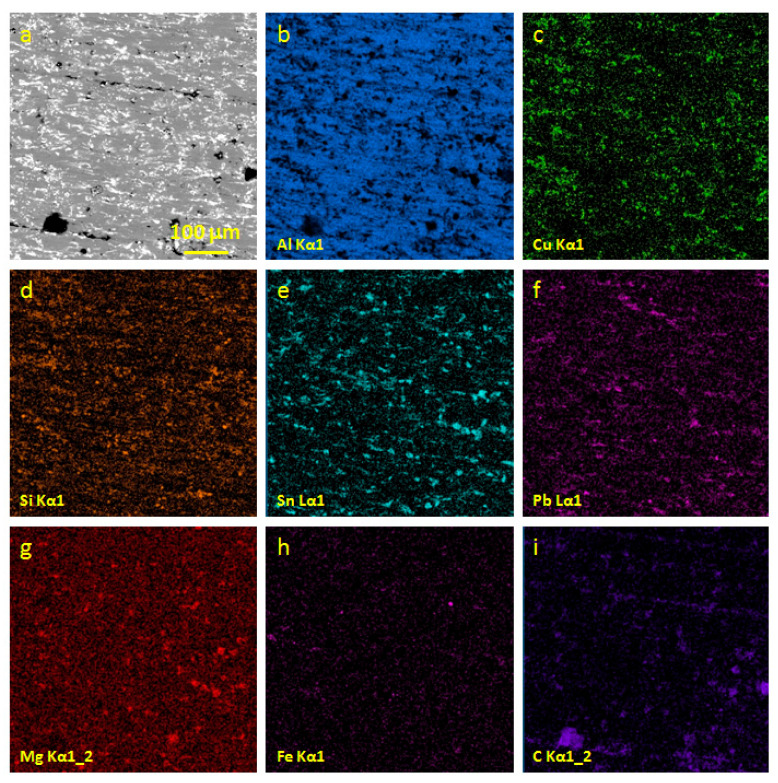
The friction surface of the alloy AO-5.8 (**a**); maps of the distribution of aluminum (**b**); copper (**c**); silicon (**d**); tin (**e**); lead (**f**); magnesium (**g**); iron (**h**) and carbon (**i**).

In the process of friction, alloys undergo significant changes in the structure and composition of the friction surface. The difference between the surface before friction and the surface after friction is illustrated in Figure 2 and Figure 3. We assume that the main changes in the composition of the friction surface occur at the initial stage of running in, with further friction, the composition of the surface (secondary) structures does not change significantly. Table 3 shows the compositions of the secondary structures on the friction surfaces of the antifriction aluminum alloys. Table 4 shows the tribological characteristics of the antifriction aluminum alloys.

Theoretical analysis was carried out in terms of the highest self-organization probability. As a result of self-organization, the secondary structures are formed. Processes in the secondary structures result in sharp decrease in wear rate and increase in seizure load. It has been shown that for the highest self-organization probability it is necessary that relatively heavy elements should be transferred to the friction zone with seizure, while relatively light elements should be transferred to the friction area without seizure. Thus, the likelihood of seizure increases with decrease seizure load and with decreased content of heavy elements in secondary structures.

The friction surfaces that work with seizure have increased wear rate. Therefore, it could be expected that alloys with increased content of heavy elements within the secondary structure composition will have relatively low wear rate. Heavy elements include elements heavier than aluminum.

The secondary structures of the AO-8.7 alloy have the lowest content of heavy elements (total Cu + Pb + Sn + Zn = 7.3%) (Table 3). Therefore, despite the high value of the seizure load, when the AO-8.7—steel pair is tested, the probability of seizure increases. This is evidenced by the highest iron content in the secondary structures of the said alloy. As a result, the AO-8.7 alloy has the highest wear.

The seizure load of the AO-5.8 alloy is slightly less than the seizure load of the alloy AO-8.7. The content of heavy elements in the secondary structures of the AO-5.8 alloy (9.9%) is significantly higher than the content of heavy elements in the secondary structures of the AO-8.7 alloy. Therefore, the friction of a pair of AO-5.8—steel occurs almost without seizure. It is evidenced by low iron content (Table 3) in the secondary structures. In such alloys (provided that they work without seizure), preferable mass transfer of relatively light elements into the friction area is desired for reducing the wear. In the secondary structures of the said alloy the sum of light elements (lighter than Al) (C + Si + O) is the largest one (50.2%) (Table 3). The alloy AO-5.8 has the lowest wear (Table 4). Typical area of the AO-5.8 friction surface is shown in Figure 3. 

The figures show that the tin content on the friction surface demonstrates almost 2-fold decrease compared to the initial structure. In the initial structure, tin is located over the grain boundaries. On the friction surface, it is more evenly distributed. Carbon appears on the surface only after friction and, basically, occupies the tin-free areas.

The AO-11 and AO-7.6 alloys have low seizure loads (Table 4), i.e., they without secondary structures are prone to seizure in the wear testing process. The secondary structures of the AO-7.6 alloy comprise the largest amount of heavy elements (11.9%). Therefore, the AO-7.6 alloy is not prone to seizure in the wear test process in the presence of secondary structures. It is proved by relatively low iron content in the secondary structures. With normal friction (without seizure), preferably light elements should be transferred into the friction zone to decrease the wear. The secondary structures of the AO-7.6 alloy comprise 38.4% of light elements (with their larger amount only in the secondary structures of the AO-5.8 alloy). In this connection, the wear of the AO-7.6 alloy is marginally higher than that of the AO-5.8 alloy. The secondary structures of the AO-11 alloy comprise 10.3% of heavy elements (Table 3), that, probably, is sufficient to prevent seizure development. It is proved by relatively low iron content in the secondary structures. The light element content in the secondary structures of the AO-11 alloy demonstrates the 1.4-fold decrease compared to the light element content in the secondary structures of the AO-7.6 alloy. It may explain increased wear of the AO-11 alloy in the process of friction without seizure.

The AO-5.4 and AO-9.8 alloys have the highest seizure load among all of the tested alloys. Therefore, they are least prone to seizure. It is proved by relatively low iron content in the secondary structures of the alloys. With normal friction without seizure, preferable transfer of light elements in the friction zone is desired. The light element content in the secondary structures of the AO-5.4 alloy demonstrates the 1.5-fold increase compared to their content in the secondary structures of the AO-9.8 alloy. In the secondary structures of the AO-9.8 alloy, the light element content is the lowest one. This is due to the fact that the wear of the AO-9.8 alloy is 1.4 times higher than the wear of the AO-5.4 alloy.

The AO-9.6 alloy has a comparably low seizure load (Table 4). The secondary structures of the AO-9.6 alloy comprise less than 10% of heavy elements (9.9%). The AO-9.6 alloy is prone to seizure, though to a lesser extent compared to the AO-8.7 alloy. This is evidenced by increased iron content and manganese presence in the secondary structures. A comparably low content of light elements in the secondary structures of the AO-9.6 alloy (31.9%) results in increased wear in the locations of friction without seizure. The wear value for the AO-9.6 alloy considerably exceeds the wear value of the tested alloys, except for the AO-8.7 alloy. The 9.6 alloy wears the steel most intensively.

The AO-6.4 has a high seizure load, while its secondary structures comprise more than 10% of heavy elements (11%). For this reason, the AO-6.4 alloy is not prone to intensive seizure. The secondary structures of the AO-6.4 alloy comprise the small amount of light elements (23.3%), so the alloy is subjected to a noticeable wear in the friction zones without seizure.

## 4. Conclusions

To describe the relationship between the composition of secondary structures and the wear of antifriction aluminum alloys non-equilibrium thermodynamics and self-organization theory have been applied. It is shown that in order to reduce the wear rate as a result of self-organization, it is necessary that mainly heavy elements are transferred to the friction zones with seizure, and predominantly light elements to the friction zones without seizure. Qualitative analysis has been carried out to determine the relationship between the secondary structure compositions of the alloys and their tribilogical characteristics. It has been shown that the wear rate of the antifriction aluminum alloys depends not only on alloy compositions but also on compositions of their secondary structures. Consequently, when developing the alloys, it is necessary to take compositions of the formed secondary structure into consideration.

## Figures and Tables

**Table 1:** Steel composition.

Content of Elements, %wt												
Main Components							Additives					
**Ni**	**Cr**	**Mn**	**Si**	**C**	**Mo**	**Fe**	**Cu**	**P**	**S**	**Al**	**V**	**Nb**
2.81	0.681	0.625	0.295	0.35	0.477	94.42	0.087	–	0.011	0.018	0.185	0.013

**Table 2:** Chemical compositions of antifriction aluminum alloys.

No	Alloy	Content of Elements, %wt							
		Sn	Pb	Cu	Zn	Mg	Si	Ti	Al
1	AO 11	11.0	2.6	3.9	2.6	–	0.1	0.01	79.8
2	AO 9.8	9.8	2.5	4.5	2.4	1.2	0.6	0.03	79.0
3	AO 9.6	9.6	3.2	4.9	4.4	0.3	0.1	0.02	77.5
4	AO 8.7	8.7	3.2	3.4	2.9	0.4	0.5	0.03	80.9
5	AO 7.6	7.6	3.3	4.0	0.5	0.07	1.0	0.06	83.5
6	AO 6.4	6.4	3.0	4.1	1.9	1.4	0.9	0.01	82.3
7	AO 5.8	5.8	2.7	4.1	2.3	1.5	1.5	0.03	82.1
8	AO 5.4	5.4	2.6	3.5	2.3	1.7	0.8	0.03	83.7

**Table 3:** Compositions of secondary structures on friction surfaces of antifriction aluminum alloys.

No	Alloy	Content of Elements, %wt																	
		Sn	Pb	Cu	Zn	Mg	Si	Cr	Al	C	O	Na	Cl	Ca	K	S	P	Fe	Mn
1	AO-11	2.7	0.9	4.7	2.0	0.1	0.3	–	60.2	17.0	11.4	0.1	0.1	0.1	–	–	–	0.3	–
2	AO-9.8	3.2	0.5	4.5	1.8	0.6	1.3	–	68.0	11.3	8.1	–	–	–	–	0.2	–	0.3	–
3	AO-9.6	2.2	0.7	3.6	3.4	0.2	0.2	–	56.9	18.1	13.6	0.3	0.2	0.1	0.1	–	–	0.4	0.1
4	AO-8.7	1.9	0.6	2.8	2.0	0.1	2.5	0.1	50.8	21.9	15.1	0.3	0.1	0.1	0.1	–	–	1.6	–
5	AO-7.6	3.6	1.3	5.4	0.9	0.3	9.1	–	49.3	17.0	12.3	–	0.1	0.2	–	–	0.1	0.3	–
6	AO-6.4	3.7	0.6	4.8	1.9	0.8	1.6	–	64.1	12.8	8.9	–	0.1	0.1	–	0.1	0.1	0.4	–
7	AO-5.8	3.2	2.6	1.9	1.4	0.4	0.5	–	39.7	34.6	15.1	–	–	0.1	–	–	–	0.2	–
8	AO-5.4	2.8	0.9	3.3	1.8	0.6	2.1	–	59.9	18.6	9.7	–	–	0.1	–	–	–	0.2	–

**Table 4:** Tribological characteristics of antifriction aluminum alloys.

Alloy Name	Seizure Load, N	Wear of Material, mg	Wear of Steel, mg
AO-11	1650	1.2	0.6
AO-9.8	2832	0.7	0.7
AO-9.6	2107	2.0	2.1
AO-8.7	2407	2.4	0.8
AO-7.6	1823	0.5	0.8
AO-6.4	2767	0.9	1.0
AO-5.8	2330	0.4	0.6
AO-5.4	2845	0.5	0.7
